# Dose-dependent effects of a genistein-enriched diet in the heart of ovariectomized mice

**DOI:** 10.1007/s12263-012-0323-5

**Published:** 2012-10-30

**Authors:** Ba Tiep Nguyen, Georgios Kararigas, Hubertus Jarry

**Affiliations:** 1 Department of Endocrinology, Goettingen University Hospital, Robert-Koch-Str. 40, 37075 Goettingen, Germany; 2 Institute of Gender in Medicine and Center for Cardiovascular Research, Charite University Hospital, Hessische Str. 3-4, 10115 Berlin, Germany; 3 Present Address: Faculty of Veterinary Medicine, Hanoi University of Agriculture, Hanoi, Vietnam

**Keywords:** Cardiac mass, Diet, Genistein, Soy

## Abstract

The isoflavone genistein is used as a pharmacological compound and as a food supplement. The duration and the level of exposure of humans to genistein are considerable. However, the magnitude of genistein-supplemented dietary interventions necessary to induce any changes in the heart has not been studied so far. The aim of this study was to investigate the dose-dependent effects of dietary genistein in the disease- and stress-free mouse heart. Female C57BL/6J mice at the age of 2 months were ovariectomized and randomly assigned to feed on diets with seven different genistein doses (0.01, 0.03, 0.1, 0.3, 1, 3 and 10 g genistein/kg food) for 3 months. Mice with intact ovaries or ovariectomized fed on soy-free diets were used as controls. Ovariectomy led to an increase in body weight, while the two highest genistein doses prevented this increase. Absolute uterus weight was decreased in the ovariectomized group and all genistein groups except for the 10 g/kg food group compared with the intact ovaries/soy-free group. Considering cardiac mass, although the 3 and 10 g/kg food groups had significantly lower absolute heart weight than all other groups, heart-to-body-weight ratios did not differ between these two groups and the intact ovaries/soy-free group, while all remaining groups had smaller ratios. Next, we observed dose-dependent effects of genistein on cardiac gene expression. The present findings indicate that exposure of female mice to the soy isoflavone genistein influences body weight and cardiac mass and gene expression in a dose-dependent manner. Human exposure to dietary genistein supplements may influence cardiac function.

## Introduction

Cardiovascular disease (CVD) represents a major cause of morbidity and mortality in the developed world (Lakatta 2002; Yusuf et al. 2001). In women, the incidence of CVD rises substantially with menopause. Due to this, it has been generally believed that the loss of oestrogen at menopause might be a major contributing factor to the increased risk for CVD. Consequently, several combinations of hormone therapies with naturally occurring and/or synthetic products have been used widely. However, unexpected negative findings from large randomized clinical trials (Anderson et al. 2004; Rossouw et al. 2002) and conflicting results from animal studies have led to controversy about the use of hormone therapies.

Along this line, soy-rich foods have been studied extensively for their ability to reduce cholesterol levels (Zhan and Ho 2005). Epidemiological studies suggest that the coronary benefits of soy extend beyond lipid lowering (Zhang et al. 2003). Soy is a rich source of isoflavones and phytoestrogens (Murphy et al. 2002). Genistein (GEN) is an isoflavone derivative found in plants, which has been shown to inhibit tyrosine kinases (Akiyama et al. 1987) and could, therefore, lead to detrimental effects in the heart (Sereno et al. 2008; Force et al. 2007) and to interact with oestrogen receptors (Davis et al. 1999). The latter activity of GEN has led to its use as a substitute of oestrogen in hormone therapy regimes. However, GEN is not only used as a pharmacological compound but also as a food supplement. Therefore, the duration and the level of exposure of humans to GEN are considerable.

The effects of GEN have been generally studied in the classical target organs of oestrogen, such as mammary gland, uterus and bone. However, little is known about the potential role of dietary GEN directly in the disease- and stress-free heart. In the present study, we investigated the dose-dependent effects of dietary GEN in the hearts of female C57BL/6J mice. We tested the hypothesis that GEN will affect body weight, cardiac mass and gene expression in a dose-dependent manner.

## Materials and methods

### Animals and diets

**Fig. 1:**
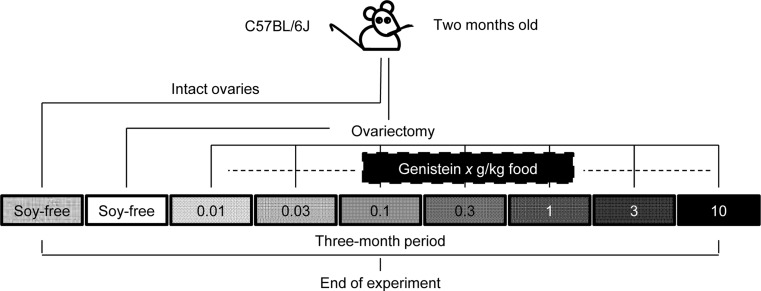
Study design. Female C57BL/6J mice were ovariectomized and fed diets with seven different GEN doses for 3 months. Mice with intact ovaries or ovariectomized fed on soy-free diets were used as controls

Female C57BL/6J mice (Winkelmann, Borchen) at 2 months of age were ovariectomized (OVX) and randomized into seven groups receiving a genistein-enriched diet (Ssniff, Soest) at a concentration of 0.01, 0.03, 0.1, 0.3, 1, 3 and 10 g/kg food, respectively. An OVX group and another with intact ovaries both receiving a soy-free diet were used as controls. The experimental design is shown in Fig. 1. All mice were fed with regular rodent diet containing soy after weaning. Immediately after OVX, mice were fed with a soy-free diet, enriched with or without GEN. The mice were kept on a 12:12-h light/dark cycle in temperature-controlled rooms with water ad libitum. All mice were monitored throughout the study, and no mouse showed signs of intoxication or health impairment. After 3 months, the mice were killed under CO_2_ anaesthesia. Following body and organ weight measurements, organs were snap frozen in liquid nitrogen and stored at −80 °C until RNA isolation. All experiments were approved by the Landesamt für Verbraucherschutz, Braunschweig, Germany.

### Quantitative real-time RT-PCR

Total RNA was isolated from left ventricles using the RNeasy Total RNA Kit (Qiagen, Hilden). cDNA was synthesized using the M-MLV Reverse Transcriptase (Promega, Madison) and random primers (Invitrogen, Karlsruhe). Reactions were performed as described previously (Kararigas et al. 2011; Nguyen et al. 2012) using SYBR Green (Applied Biosystems, Foster City). For *Myocd* analysis, we used the Mm_Myocd_1_SG primers, for *Esr1* the Mm_Esr1_2_SG primers and for *Esr2* the Mm_Esr2_1_SG primers; all from the QuantiTect Primer Assay (Qiagen, Hilden). For *Igf1* analysis, we used the following: forward primer 5′-CTTCAACAAGCCCACAGGCTA-3′, reverse primer 5′-GCTCCGGAAGCAACACTCAT-3′ and probe 5′-CTCCAGCATTCGGAGGGCACCTC-3′.

### Statistical analysis

All data were analysed statistically using the R version 2.14.2 software. Data are shown as the mean ± SEM. Comparisons between multiple groups were performed using analysis of variance with Tukey’s post hoc test adjusting for multiple comparisons, considering *P* ≤ 0.05 significant.

## Results

### Study design

In the present study, we investigated the dose-dependent effects of GEN in the hearts of OVX mice under physiological conditions. We tested the hypothesis that GEN will affect body weight, cardiac mass and gene expression in a dose-dependent manner. For this purpose, we selected seven concentrations of GEN to include a wide range of GEN levels with relevance to human consumption and doses of GEN that have been previously shown to exert oestrogenic-like effects in rodents (Nguyen et al. 2012). Mice that received GEN were ovariectomized and a further OVX group on soy-free diet was used as one of two controls. The second control group was soy-free-fed mice with intact ovaries (Fig. 1).

### Food consumption and actual GEN intake

We verified the extent of food consumption in all groups and asked whether an enriched diet with different amounts of GEN would affect the intake of food. Based on own previous experience and the literature, we had expected an average intake of 3 g food per mouse per day. We found that the amount of food consumption in the groups of 3 and 10 g GEN/kg food was significantly decreased compared with the food consumption of all remaining groups, where no significant effect on average food consumption was observed (Table 1). Considering the average food consumption, we were able to calculate the actual GEN intake per mouse in each group (Table 1).

**Table 1:** Food consumption and actual GEN intake

	Intact SF	OVX SF	GEN 0.01	GEN 0.03	GEN 0.1	GEN 0.3	GEN 1	GEN 3	GEN 10
Food consumption (g/mouse/day)	3.39	3.27	3.19	3.27	3.35	3.33	3.18	2.86^a^	2.82^a^
Actual GEN intake (mg/mouse/day)	–	–	0.032	0.098	0.36	0.99	3.18	8.58	28.2

^a^ *P* < 0.05 versus all groups

### Dose-dependent effects of dietary GEN on body weight

**Fig. 2:**
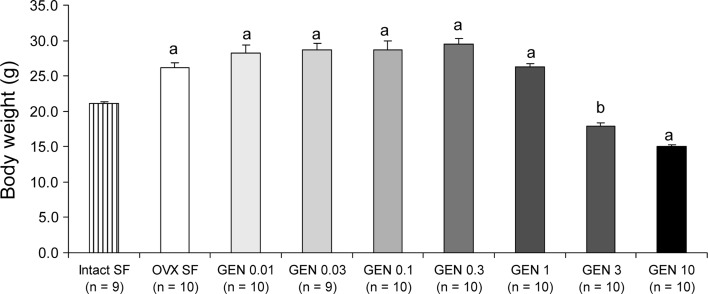
Dose-dependent effects of GEN on body weight. Ovariectomy led to an increase in body weight, while the two highest GEN doses prevented this increase. Increased body weight was observed in all remaining GEN groups compared with the intact SF group. *a* *P* < 0.001 and *b* non-significant versus intact SF

As it was expected, the removal of the ovaries led to an increase in body weight when compared to mice with their ovaries intact (24 % increase; *P* < 0.001) (Fig. 2). However, the two highest concentrations of GEN, that is, 3 and 10 g/kg food, were able to inhibit body weight gain (Fig. 2). In fact, the group of mice fed on the diet with the highest GEN content had a significantly lower body weight than the group of mice with intact ovaries (29 % lower; *P* < 0.001) (Fig. 2). On the other hand, the remaining doses of GEN did not have any major effect on body weight (Fig. 2).

### Dose-dependent effects of oral GEN treatment on uterus and heart weight

**Fig. 3:**
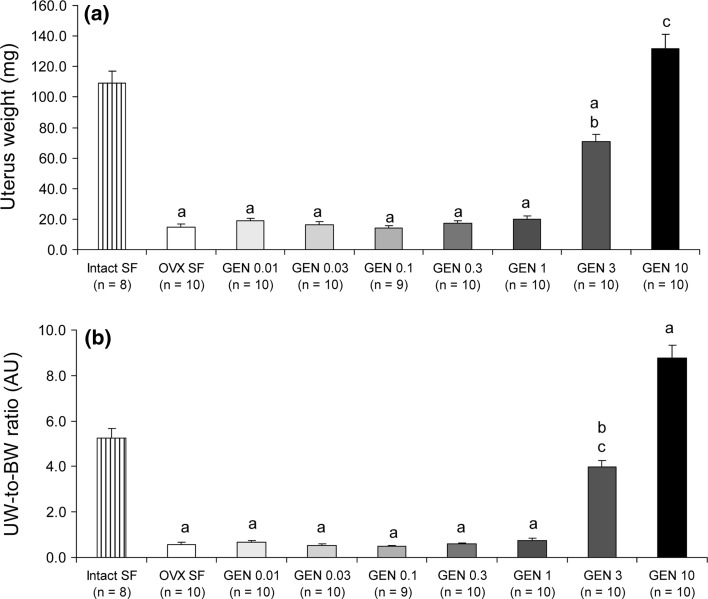
Dose-dependent effects of GEN on uterus weight. **a** Absolute uterus weight was decreased in the OVX group and all GEN groups except for GEN 10 compared with the intact SF group. **b** Uterus-to-body-weight ratio showed the same pattern. *a* *P* < 0.001 and *c* 0.05 versus intact SF; *b* *P* < 0.001 versus OVX SF

Next, we assessed the weight of the uterus of mice in the different groups. As expected, we found that OVX mice had a significant decrease in uterus weight compared with mice with intact ovaries (7.5-fold decrease; *P* < 0.001) (Fig. 3a). In contrast, the GEN 10 g/kg food group had significantly higher uterus weight than the intact ovaries/soy-free-fed group (21 % higher; *P* < 0.05) (Fig. 3a). On the other hand, although the dose of 3 g GEN/kg food led to a significant increase in uterus weight compared with all remaining GEN groups, the uterus weight of this group was significantly lower than that of mice with intact ovaries (1.54-fold; *P* < 0.001) (Fig. 3a). All these differences were still significant even after relating uterus weight to body weight (Fig. 3b). The concentration of GEN 1 g/kg food or lower did not exert major effects on uterus weight.

**Fig. 4:**
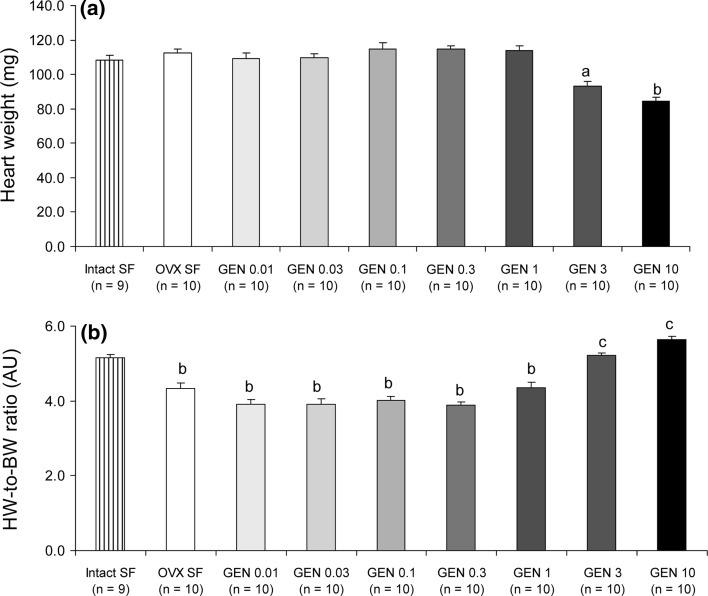
Dose-dependent effects of GEN on heart weight. **a** Although GEN 3 and 10 had significantly lower heart weight than all other groups, **b** heart-to-body-weight ratios did not differ between these two groups and the intact SF group, while all remaining groups had smaller ratios. *a* *P* < 0.01, *b* 0.001 and *c* non-significant versus intact SF

On the other hand, the effects of GEN on heart weight were more complex. In particular, the absolute heart weight of mice fed on the two highest GEN concentrations, that is, 3 and 10 g/kg food, was significantly lower when compared with all remaining groups (Fig. 4a). However, when considering the heart weight related to body weight, we discovered that there was no significant difference between the intact ovaries/soy-free-fed control group and the 3 and 10 g GEN/kg food groups (Fig. 4b). On the other hand, OVX and all other GEN-fed mice had significantly lower heart-to-body-weight ratios (Fig. 4b).

### Dose-dependent effects of dietary GEN on gene expression in the heart

**Fig. 5:**
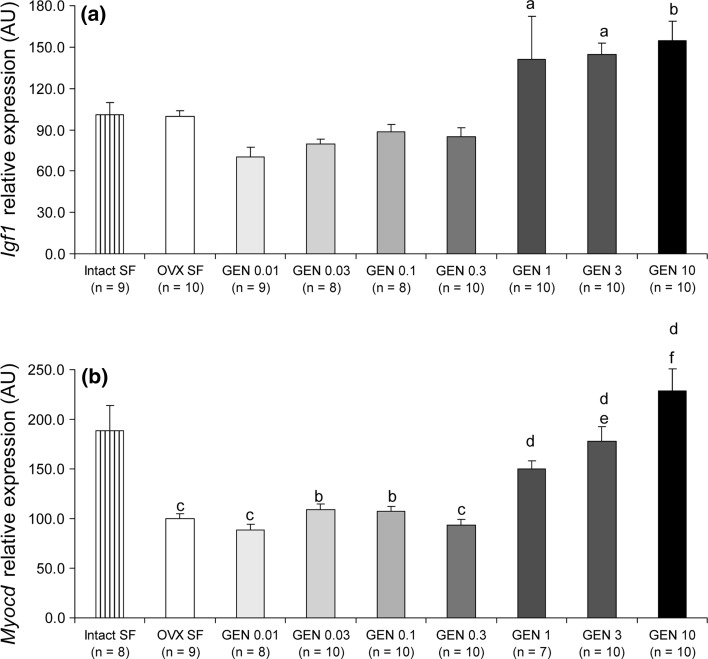
Dose-dependent effects of GEN on cardiac gene expression. GEN influenced the expression of **a** *Igf1* and **b** *Myocd* in a dose-dependent manner. *a* *P* < 0.05, *b* 0.01, *c* 0.001 and *d* non-significant versus intact SF; *e* *P* < 0.01 and *f* 0.001 versus OVX SF

**Fig. 6:**
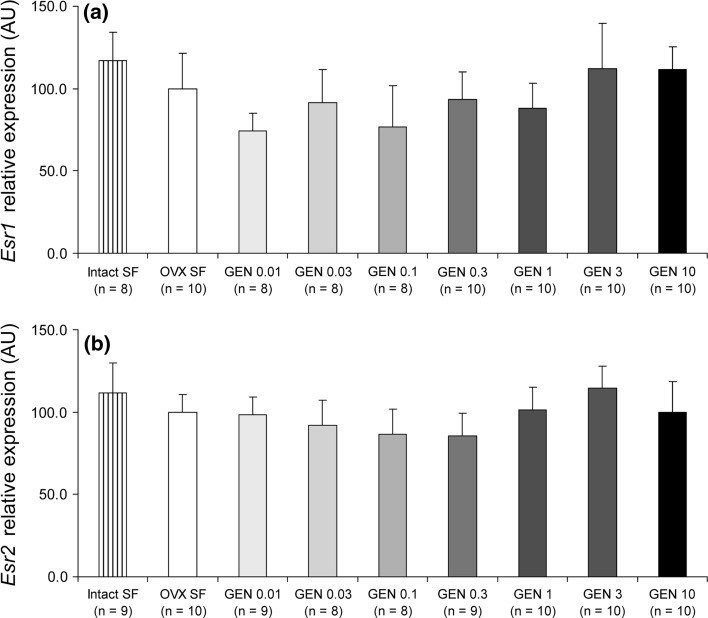
Left ventricular expression of oestrogen receptors. Expression levels of *Esr1* (**a**) and *Esr2* (**b**) were similar in all treatment groups

Following the observed effects of GEN on body and organ weight, we assessed the role of GEN on cardiac gene expression. In particular, we studied the expression of insulin-like growth factor 1 (*Igf1*) and myocardin (*Myocd*) in the mouse left ventricle with real-time RT-PCR. We chose *Igf1*, because it is involved in Akt signalling protecting cardiomyocytes against injury (Fujio et al. 2000), and *Myocd* as it has been previously shown to be under nuclear hormone receptor regulation (Li et al. 2007). We found that the GEN 1, 3 and 10 g/kg food groups of mice had the highest expression of *Igf1* (Fig. 5a). Although in the remaining GEN groups, there was a lowering trend in the expression of *Igf1*, no statistical significance was reached. On the other hand, the expression levels of *Myocd* were significantly lower in the OVX and the GEN 0.01–0.3 g/kg food groups than the intact ovaries/soy-free-fed control group (Fig. 5b). In contrast, the expression of *Myocd* in the 1–10 g GEN/kg food was not statistically different from that in the intact ovaries/soy-free-fed group (Fig. 5b). Following these differences, we verified the expression levels of both oestrogen receptor subtypes. We found that their expression was similar in all groups (Fig. 6).

## Discussion

In the present study, we investigated the dose-dependent effects of GEN in the heart of disease- and stress-free mice. We employed seven different concentrations of GEN in OVX mice and found that significant effects were exerted on body and organ weight and cardiac gene expression by the three highest GEN concentrations.

In particular, we discovered that although the average daily food intake per mouse was comparable in most groups, mice fed on a diet with the two highest GEN doses exhibited a significant decrease in food intake. While this was a surprising finding and there is no obvious explanation for this difference, we anticipate that GEN might exert direct effects on the brain, which in turn has a major role in the control of food intake (Del Parigi et al. 2002). Subsequently, we found that OVX mice had an increase in their body weight compared with mice with intact ovaries. However, this increase was hindered in the GEN 3 and 10 g/kg food groups, which may be the result of the combination of lower food intake in these groups and unrecognized molecular pathways involved in the control of body weight and adiposity signals that may be regulated by high GEN levels. In fact, it has been suggested that controlling meal size may be relevant for the development of efficacious therapeutic tools to reduce eating (Lutz 2006). Along this line, the leptin receptor long form and the SH2-tyrosine phosphatase Shp2 might be potential targets of GEN in the brain, whose modulation by leptin and oestrogen signalling regulates food intake and energy balance (He et al. 2012; Ring and Zeltser 2010). Based on our findings, we propose that GEN supplementation of a well-balanced healthy diet might be instrumental in whole body weight loss approaches. However, deciphering the underlying molecular pathways holds the promise for the development of novel therapeutic means.

Assessing absolute heart weight, we found that mice fed on the two highest GEN doses had significantly smaller hearts than any other GEN group. This finding associated with the effects on body weight fits the obvious model that a bigger body requires a bigger heart and vice versa. However, heart-to-body-weight ratios revealed that there were no significant differences between the groups with the two highest GEN concentrations and the intact ovaries/soy-free-fed control group. This suggests that in the absence of endogenous hormones and particularly oestrogen as a result of ovariectomy, GEN might be crucial for the maintenance of cardiac stability. Preserving the size and structure of an organ would be of utmost importance for the organ’s function. Therefore, we believe that GEN supplementation of a normal diet may be beneficial for cardiac function, especially in postmenopausal women. However, this hypothesis needs to be tested in a study where cardiac function is also assessed.

On the other hand, it should be taken into account that hormonal actions in the heart may be sex specific and not always beneficial (Kararigas et al. 2010, 2012). In fact, in a genetic model of hypertrophic cardiomyopathy, it was shown that a soy-based diet was beneficial in females but harmful in males (Luczak et al. 2011). The observed beneficial effects in females were postulated to be attributed to increased Igf1 pathway expression (Luczak et al. 2011). Downstream activating signalling of Akt by Igf1 in cardiomyocytes is protective against injury (Fujio et al. 2000). To this extent, premenopausal women have been shown to have significantly higher levels of nuclear-localized phosphorylated Akt in cardiomyocytes compared with age-matched men or postmenopausal women (Camper-Kirby et al. 2001). In the present study, we found that the three highest doses of GEN, that is, 1, 3 and 10 g/kg food, led to increased *Igf1* expression compared with both control groups. Based on these findings, we put forward that the GEN-induced *Igf1* expression is beneficial for the heart. However, considering sexual dimorphism in hormonal effects, it would be very interesting to verify the role of GEN in the disease- and stress-free heart of male mice.

The potential use of GEN as a natural selective oestrogen receptor modulator (SERM) seems to be promising. In long-term hormone therapy regimens, the use of oestrogen may exert negative effects in the breast and the endometrium. However, a clear advantage of GEN is that it may behave as an oestrogen receptor antagonist in both of these tissues. Along this line, in a previous study, we did not observe any oestrogenic-like effects of GEN in the uterus, as assessed by the expression of the insulin-like growth factor 1 (*Igf1*) gene (Nguyen et al. 2012). This indeed supports the notion that genistein is not an oestrogen receptor agonist in the uterus, whose inner cell layer is the endometrium. In addition, the incidence of uterine dysplasia was low in ovariectomized rats fed GEN, suggesting a weak oestrogen receptor agonist role of GEN also in the rat uterus (Aidoo et al. 2005).

In conclusion, we have identified dose-dependent effects of dietary GEN on body weight and directly on cardiac mass and gene expression. Further research is required to study GEN effects on cardiac function and to elucidate the molecular factors mediating these effects.

## Abbreviations

CVD: Cardiovascular disease
GEN: Genistein
OVX: Ovariectomized
